# A deep learning based assisted analysis approach for Sjogren’s syndrome pathology images

**DOI:** 10.1038/s41598-024-75925-w

**Published:** 2024-10-21

**Authors:** Peihe Jiang, Yi Li, Chunni Wang, Wei Zhang, Ning Lu

**Affiliations:** 1 School of Physics and Electronic Information, Yantai University, Yantai, 264005 China; 2 Department of Radiation Oncology, Shandong Cancer Hospital and Institute, Shandong First Medical University and Shandong Academy of Medical Science, Jinan, 250117 China; 3 State Key Laboratory of Integration and Innovation of Classic Formula and Modern Chinese Medicine, Lunan Pharmaceutical Group Co. Ltd., Linyi, 276005 China; 4 Medical Integration and Practice Center, Cheeloo College of Medicine, Shandong University, Jinan, 250012 China; 5 Pathology Department, Yantaishan Hospital, Yantai, 264003 China

**Keywords:** Deep learning, Attention mechanism, IoU loss function, Sjogren’s Syndrome, Image processing, Machine learning

## Abstract

Diagnosing Sjogren’s syndrome requires considerable time and effort from physicians, primarily because it necessitates rigorously establishing the presence lymphatic infiltration in the pathological tissue of the labial gland. The aim of this study is to use deep learning techniques to overcome these limitations and improve diagnostic accuracy and efficiency in pathology. We develop an auxiliary diagnostic system for Sjogren’s syndrome. The system incorporates the state-of-the-art object detection neural network, YOLOv8, and enables the precise identification and flagging of suspicious lesions. We design the multi-dimensional attention module and S-MPDIoU loss function to improve the detection performance of YOLOv8. By extracting features from multiple dimensions of the feature map, the utilization of the multi-dimensional attention mechanism enhances the feature interaction across disparate positions, enabling the network to proficiently learn and retain salient cell features. S-MPDIoU introduces an angle penalty term that efficiently minimizes the diagonal distance between predicted and ground truth boxes. Additionally, it incorporates a flexible scale factor tailored to different size feature maps, which balances the issue of sudden gradient decrease during high overlap, thereby accelerating the overall convergence rate. To verify the effectiveness of our methods, we create a dataset of lymphocytes using labial gland biopsy pathology images collected from YanTaiShan hospital and trained the model with this dataset. The proposed model is assessed using standard metrics like precision, recall, mAP. The improved model achieves an increase in recall by 9.1%, mAP.5 by 3.2%, and mAP.95 by 2%. The study demonstrated deep learning’s potential to analysis pathology images, offering a reference framework for the application of deep learning technology in the medical domain.

## Introduction

Sjogren’s syndrome (SS) is a chronic inflammatory autoimmune systemic disease characterized by lymphocyte proliferation and progressive damage to exocrine glands^1^. In their daily work, physicians need to examine each pathological section under different magnification lenses to diagnosis Sjogren’s syndrome. This process is lengthy and time-consuming. Due to the subjective heterogeneity of physicians at different levels, misdiagnosis and missed diagnosis often occur. Accurate and efficient pathological diagnosis has become a significant challenge. Currently, the rapid development of digital pathology technology has given rise to AI-assisted pathological diagnosis. In the pathological diagnosis of lung, breast, thyroid, and other malignancies, AI-assisted diagnosis offers remarkable efficiency, stability, and reproducibility. Its performance is essentially comparable to that of professional physicians, providing a viable and promising solution for addressing the challenges inherent in manual pathological diagnosis. However, the application of AI technologies in pathological image diagnosis faces numerous technical challenges, including complexity of pathological patterns, model overfitting and generalization issues, computational inefficiency, lack of model interpretability. Addressing these challenges necessitates innovative solutions such as robust AI algorithms capable of capturing subtle pathological differences, efficient computational approaches to handle large images. Overcoming these technical barriers is crucial for the successful deployment of AI-based diagnostic tools in pathology and their acceptance by physicians and patients alike.

**Fig. 1:**
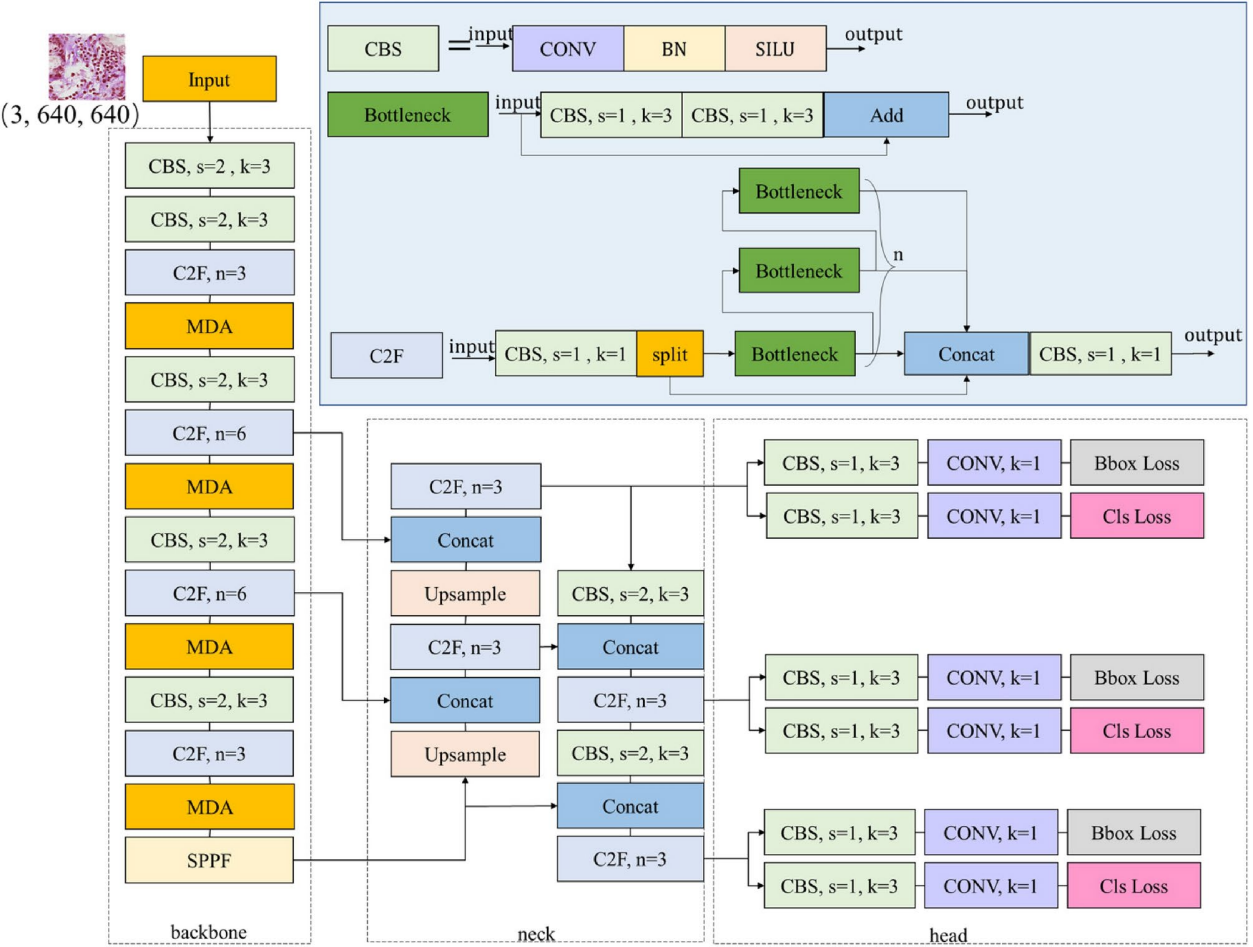
Improved YOLOv8 framework. YOLOv8 consists of three primary parts: backbone, neck, and head. “CBS” denotes “Conv + Batch Normalization + Silu”. “C2f” denotes the C2f module in YOLOv8. To enhance feature extraction from the input image, we introduce the MDA module in the backbone to assist in processing and analysis.

We employ the cutting-edge YOLOv8 model to address the challenges inherent in traditional manual diagnosis. Specifically, to tackle the difficulties arising from the small size of lymphocytes and their difficulty in being distinguished, this paper introduces two improvements to the YOLOv8 model. The improved YOLOv8 network framework is shown in Fig. 1. YOLOv8 represents a significant leap forward in object detection technology, is characterized by its sophisticated design that integrates cutting-edge backbone and neck architectures with an innovative anchor-free split head. Specifically, it utilizes a CSPDarknet53 backbone, which is built upon the Darknet53 network and enhanced with cross-stage partial connections (CSP) for improved feature extraction while maintaining computational efficiency. The neck of the network incorporates feature fusion techniques, such as Spatial Pyramid Pooling (SPP) and Path Aggregation Network (PAN), to aggregate multi-scale features, enhancing the model’s robustness and detection capabilities. The loss function of YOLOv8 comprises both bounding box loss and classification loss. The bounding box loss is a composite of CIoU loss and DFL loss, designed to optimize the localization of objects by considering factors such as intersection over union (IoU), aspect ratio, and distance from the center point. Meanwhile, the classification loss employs BCE loss to accurately classify the detected objects.

The main contributions of this paper are as follows:

1. An auxiliary diagnostic system is proposed for Sjogren’s syndrome based on this model, effectively improving diagnostic efficiency.
2. A novel S-MPDIoU loss function is designed to replace the original CIoU loss function, accelerating network training and improving detection performance.
3. A novel attention module multi-dimensional attention (MDA) is designed and integrated into the backbone of the network to enhance the network’s ability to extract and fuse features.

The rest organized of this study is: Section 2 presents a concise overview of the relevant research work. Section 3 presents a detailed explanation of our method. Section 4 presents a detailed introduction to the experimental process and analyzes the experimental results. Section 5 presents the conclusion.

## Related work

### Relevant applications of artificial intelligence in the medical domain

Artificial Intelligence is finding widespread applications across a broad spectrum of domains, including finance, agriculture^2^, and numerous others, but it is in the medical field where its revolutionary impact is particularly pronounced. By harnessing advanced algorithms and machine learning techniques, AI has shown remarkable efficacy in facilitating the prompt diagnosis of Parkinson’s disease^3^, empowering clinicians to identify the disease’s onset at an earlier stage. This pivotal shift enables more timely interventions, leading to improved patient outcomes, slowed disease progression, more effective symptom management, and ultimately, an enhanced quality of life for those affected. Parallel to this, AI’s integration into voice pathology diagnosis has brought about a significant transformation^4^. Voice disorders, which encompass a wide spectrum from benign conditions to precursors of serious health concerns, often pose challenges in achieving accurate and prompt diagnosis. However, with the emergence of AI-powered systems, the accuracy of voice pathology diagnosis has soared. These sophisticated systems analyze vocal patterns meticulously, detecting even the most subtle changes indicative of a disorder. By enabling earlier detection, AI-based voice pathology diagnosis paves the way for more targeted treatment plans, mitigating patient suffering, and enhancing overall healthcare outcomes. Moreover, researchers like Shen et al.^5^ have pioneered innovative multi-scale convolutional neural network (CNN) architectures tailored for lung nodule classification in medical imaging. Their approach leverages the power of multi-scale feature extraction, enabling the network to capture intricate local patterns alongside broader contextual information across various resolutions of lung CT scans. Similarly, Ho et al.^6^ have further advanced breast cancer detection through a deep learning method that integrates multi-scale feature fusion, effectively combining low-level details with high-level abstractions to enhance diagnostic accuracy. As technology continues to evolve, the application of AI in medicine promises continuous improvements and enhancements, reshaping the future of healthcare and enhancing the lives of patients worldwide.

### The development of YOLO series object detection networks

Real-time object detection aims to classify and locate targets with low latency, which is crucial for practical applications. Over the past few years, significant efforts have been made to develop efficient detectors, with the YOLO series emerging as a mainstream choice. Starting with YOLOv1^7^, YOLOv2^8^, and YOLOv3^9^, a typical detection architecture was established, comprising a backbone network, neck, and head. YOLOv4^10^ and YOLOv5 introduced the CSPNet design to replace DarkNet, while incorporating data augmentation strategies, an enhanced PAN, and a broader range of model scales. The technical highlights of YOLOv6 lie in its optimized EfficientRep Backbone and Rep-PAN Neck for feature extraction, Anchor-free detection with SimOTA and SIoU for precise localization. YOLOv7^11^ introduced E-ELAN to enable rich gradient flow paths and explored various trainable bag-of-freebies methods. YOLOv8, meanwhile, proposed the C2f building block for efficient feature extraction and fusion. YOLOv9^12^, introduced GELAN to improve the architecture and PGI to enhance the training process. YOLOv10^13^ featured training without Non-Maximum Suppression (NMS), enhanced global modeling capabilities through large kernel convolutions and partial self-attention.

### Bounding box regression loss function

At the beginning of the development of object detection networks, the $\mathscr {L}_{n^-}$norm loss function was commonly used due to its simplicity. However, it exhibited sensitivity to various scales of bounding boxes. As the field evolved, the IoU-based bounding box loss function gradually replaced $\mathscr {L}_{n^-}$ norm loss function as the mainstream approach for computing bounding box loss. This is attributed to its ability to reflect the scale differences between the ground truth and predicted bounding boxes. IoU(Intersection over Union) is the ratio of the intersection area to the union area between the predicted and ground truth bounding boxes. The calculation formula is shown below, where $B^{pred}$ represents the predicted bounding box and $B^{gt}$ represents the ground truth bounding box.

1 $$\begin{aligned} IoU=\frac{B^{pred}\cap B^{gt}}{B^{pred}\cup B^{gt}} \end{aligned}$$

The main idea of IoU-based bounding box loss functions is to minimize the IoU loss. This enables the network to learn effective strategies for fine-tuning the position and size of predicted bounding box, thus ensuring precise alignment with the ground truth bounding box. In recent years, numerous studies aim to improve the IoU loss function. Given that when two boxes fail to overlap, IoU becomes zero and cannot contribute gradients. To address this problem, GIoU^14^ offers a more precise distance metric. The calculation formula for GIoU is as follows, where *C* is the smallest box covering $B^{gt}$ and $B^{pred}$, $\left| C\right|$ represents its area. However, GIoU will lost effectiveness when the predicted bounding box is completely covered by the ground truth bounding box.

2 $$\begin{aligned} GIoU=IoU-\frac{\left| C-B^{pred}\cup B^{gt} \right| }{\left| C\right| } \end{aligned}$$

DIoU^15^ introduces a novel distance penalty term that directs the predicted bounding box towards the center of the ground truth bounding box, addressing the problem of GIoU. The calculation formula for DIoU is as follows, where $\rho ^2\left( B^{pred},B^{gt} \right)$ represents the squared Euclidean distance between the centers of the two bounding boxes, and $C^2$ represents the squared diagonal length of the smallest box covering $B^{gt}$and $B^{pred}$.

3 $$\begin{aligned} DIoU=IoU-\frac{\rho ^2\left( B^{pred},B^{gt} \right) }{C^2} \end{aligned}$$

DIoU takes into account the distance between the predicted and ground truth bounding box, thus offering a more comprehensive evaluation of the detection quality. However, when the centers of the two boxes overlap, DIoU degrades to IoU. To address this problem, the CIoU^16^ loss function introduces a penalty term that accounts for the similarity of the aspect ratios, thus further enhancing the convergence performance. The formulas for CIoU are as follows, where $\nu$ measures the consistency of the aspect ratios between the two bounding boxes, and $\alpha$ is a positive trade-off parameter that balances the distance and aspect ratio.

4 $$\begin{aligned} & CIoU=IoU-\frac{\rho ^2\left( B^{pred},B^{gt} \right) }{C^2}-\alpha \nu \end{aligned}$$

5 $$\begin{aligned} & \nu =\frac{4}{\pi ^2}\left( \tan ^{-1}\frac{w^{gt}}{h^{gt}}-\tan ^{-1}\frac{w^{pred}}{h^{pred}}\right) ^2 \end{aligned}$$

6 $$\begin{aligned} & \alpha =\frac{\nu }{1-IoU+\nu } \end{aligned}$$

Based on DIoU, EIoU^17^ incorporates the absolute values of the width and height of the bounding boxes into the loss calculation, further accelerating the convergence speed. The formula for EIoU is as follows, where $\rho ^2\left( w^{pred},w^{gt} \right)$ and $\rho ^2\left( h^{pred},h^{gt} \right)$ represent the squared differences in width and height between the two boxes, respectively. $w_c^2$ and $h_c^2$ represent the squared width and height of the smallest box covering $B^{gt}$and $B^{pred}$.

7 $$\begin{aligned} EIoU = IoU-\frac{\rho ^2\left( B^{pred},B^{gt} \right) }{C^2}-\frac{\rho ^2\left( w^{pred},w^{gt} \right) }{w_c^2} -\frac{\rho ^2\left( h^{pred},h^{gt} \right) }{h_c^2} \end{aligned}$$

EIoU exhibits sensitivity to both the width and height of bounding boxes, thus effectively addressing the limitation of CIoU where the penalty term remains constantly zero when the aspect ratios of the predicted and ground truth bounding boxes are identical. This refinement ensures a more comprehensive evaluation of box similarity, ultimately leading to improved detection accuracy and faster convergence speed.

SIoU^18^ redefines the loss function by introducing angle loss, enabling the predicted bounding box to move to the axis closest to the ground truth bounding box during training. This avoids the phenomenon of the predicted bounding box wandering around the ground truth bounding box. The SIoU loss function comprises four components: angle loss, distance loss, shape loss, and IoU loss. The formula is as follows, where $\varDelta$ represents distance loss and $\Omega$ represents shape loss. Due to the complexity of its formula, a detailed elaboration is omitted for simplicity. However, SIoU faces challenges in terms of result interpretation due to its complex calculations. Furthermore, the introduction of multiple thresholds can complicate the model, potentially reducing reproducibility and increasing the difficulty in achieving consistent performance across different settings.

8 $$\begin{aligned} SIoU=IoU-\frac{\varDelta +\Omega }{2} \end{aligned}$$

MPDIoU^19^ incorporates the key ideas from the aforementioned IoU loss functions and addresses the problem where the predicted bounding box has similar aspect ratios but different width and height compared to the ground truth bounding box. The formula is as follows, where $w^2+h^2$ represents the squared diagonal length of the current detection feature map, $d_1^2$ and $d_2^2$ represent the squared distance between the top-left and bottom-right corners of the two bounding boxes, respectively.

9 $$\begin{aligned} MPDIoU=IoU-\frac{d_1^2}{w^2+h^2}-\frac{d_2^2}{w^2+h^2} \end{aligned}$$

In this paper, simulation experiments are conducted to evaluate the performance of the aforementioned IoU loss functions. To tackle the slower convergence speed observed in some cases for MPDIoU, we introduce a scale strategy and optimize its penalty term, resulting in a significant acceleration of its convergence speed. We name the novel IoU loss function as S-MPDIoU, and a detailed discussion will be presented in the subsequent section.

### Attention mechanism

The attention mechanism is a pivotal element in boosting the performance of neural networks, and its effectiveness has been proven across a diverse array of visual tasks. In the realm of computer vision, two prominent types of attention mechanisms are widely utilized: spatial attention and channel attention. Channel attention enables the model to discern crucial feature information pertaining to the object, while spatial attention aids in precisely locating the object.

The SE^20^ attention mechanism utilizes a Squeeze-and-Excitation process to extract channel-wise attention features. CBAM^21^ attention mechanism combines channel attention and spatial attention sequentially to improve the feature representation ability. GAM^22^ attention mechanism follows a similar strategy, also integrating channel and spatial attention, but with more parameters to ensure accuracy. SA^23^ attention mechanism employs channel grouping to reduce the parameters, and utilizes spatial and channel attention separately. CA^24^ attention mechanism enhances the performance by embedding positional information into channel attention. The ECA^25^ attention mechanism solves the computational overhead and redundancy introduced by the fully connected layer or complex model construction of traditional channel attention mechanisms through adaptive one-dimensional convolution. EMA^26^ attention mechanism incorporates positional information into channel attention feature extraction using directional pooling, while enriching semantic information and enhancing detection performance through cross-spatial multi-scale feature fusion.

It should be noted that although the integration of channel and spatial attention mechanisms can boost performance, it concomitantly entails an increased computational cost. Furthermore, obtaining cross channel relationships through channel reduction may potentially compromise the representation of deep features.

Overall, the advancements in attention mechanism have significantly enhanced the performance of neural networks across diverse visual tasks. Inspired by previous research, we have designed a novel and effective attention mechanism, MDA (Multi-Dimensional Attention), which will be discussed in detail in the subsequent section.

## Method

### Sjogren’s syndrome auxiliary diagnosis system

**Fig. 2:**
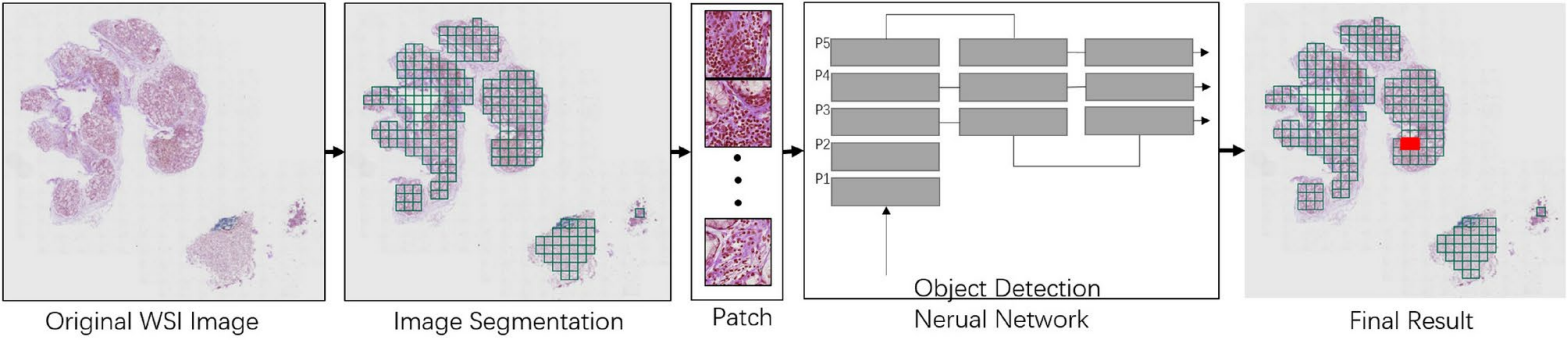
Auxiliary diagnostic system flowchart.

We train and employ a lymphocyte detection model to develop an auxiliary diagnostic system for Sjogren’s syndrome, as depicted in the flowchart in Fig. 2. Initially, the system commences by filtering out the background from the original whole slide image (WSI), subsequently extracting the pathological tissue. This extracted tissue is then systematically segmented into numerous patches, which are then individually fed into the detection model for lymphocyte identification. Upon completion of the detection process for each patch, the system consolidates and summarizes the detection results. Patches with lymphocyte detection numbers exceeding the predetermined threshold are highlighted in red, indicating potential suspicious lesions. Physicians can further confirm the presence of lymphatic infiltration based on the red markings on the system, thereby fulfilling the purpose of assisting the diagnosis.

### S-MPDIOU

**Fig. 3:**
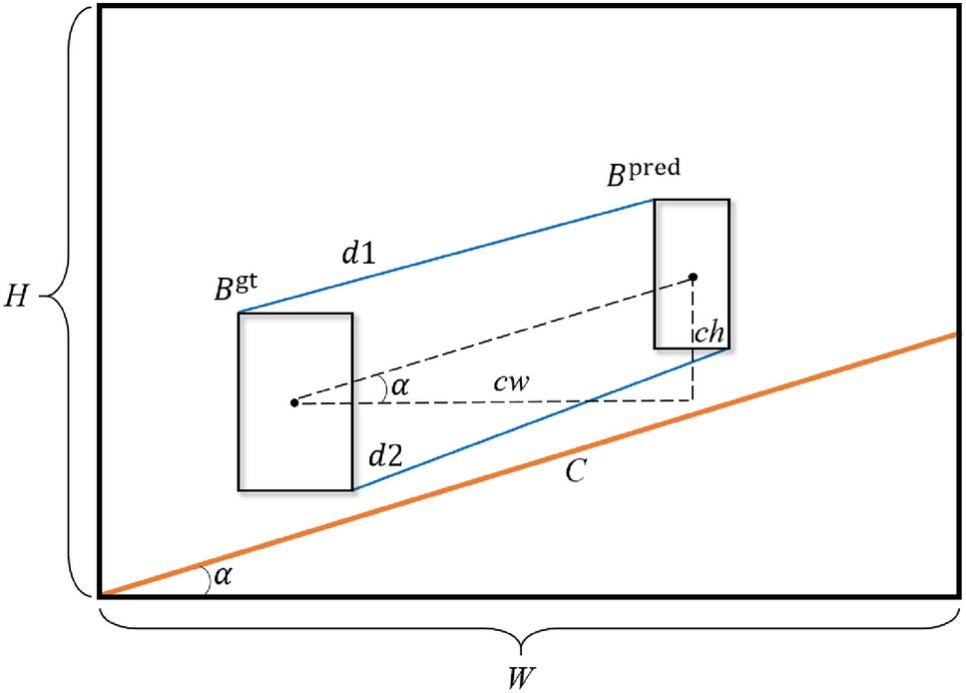
Illustration for calculating S-MPDIoU.

We propose a novel and effective IoU loss function named S-MPDIoU. The specific calculation principle of S-MPDIoU is illustrated in Fig. 3. The core calculation of S-MPDIoU primarily consists of computing the distances between the top-left and bottom-right corners of the predicted and ground truth bounding boxes. This approach enables the model to uniformly control regression metrics, such as the distance, dimension, and shape, thus avoiding errors and complexity that would otherwise arise from the separate calculation of various indicators. Additionally, the baseline *C* is calculated based on the angle between the central coordinates of two boxes, varying with the specific angles. It accelerates training and enhances detection performance.

As shown in Fig. 3, $B^{pred}$ represents the predicted bounding box, and $B^{gt}$ represents the ground truth bounding box. $\alpha$ represents the angle between the central coordinates of the two bounding boxes, which is less than $\frac{\pi }{2}$. Assuming that the central coordinates of the two bounding boxes are $\left( x_c^{pred},y_c^{pred}\right)$ and $\left( x_c^{gt},y_c^{gt}\right)$, respectively. $C_h$ and $C_w$ represent the width and height between the two centers. The coordinates of the top-left and bottom-right corners of the two bounding boxes are assumed to be $\left( x_l^{pred},y_l^{pred}\right)$, $\left( x_r^{pred},y_r^{pred}\right)$ and $\left( x_l^{gt},y_l^{gt}\right)$, $\left( x_r^{gt},y_r^{gt}\right)$. The two blue lines, *d*1 and *d*2, represent the distances between the top-left and bottom-right corners of the two bounding boxes, respectively. *H* and *W* represent the height and width of the detection feature map. The orange line *C* represents the baseline determined based on the angle between the current predicted and ground truth bounding box. The formulas for calculating each variable are as follows:

10 $$\begin{aligned} & C_h=\left| y_c^{pred}-y_c^{gt}\right| \end{aligned}$$

11 $$\begin{aligned} & C_w=\left| w_c^{pred}-w_c^{gt} \right| \end{aligned}$$

12 $$\begin{aligned} & \alpha =\tan ^{-1}\frac{C_h}{C_w} \end{aligned}$$

13 $$\begin{aligned} & d_1=\sqrt{\left( x_l^{pred}-x_l^{gt} \right) ^2+\left( y_l^{pred}-y_l^{gt} \right) ^2} \end{aligned}$$

14 $$\begin{aligned} & d_2=\sqrt{\left( x_r^{pred}-x_r^{gt} \right) ^2+\left( y_r^{pred}-y_r^{gt} \right) ^2} \end{aligned}$$

According to the definition of MPDIoU, *C* represents the diagonal of the feature map, and its calculation formula is as follows:

15 $$\begin{aligned} C=\sqrt{\left( H^2+W^2\right) } \end{aligned}$$

The definition of *C* in S-MPDIoU takes into account the relative position of the predicted and ground truth bounding box. Different *C* values are flexibly set to make the convergence speed faster compared to using a fixed *C* value. Its definition formula is shown as follows.

If $\alpha$ less than $\frac{\pi }{4}$:

16 $$\begin{aligned} w1=W,h1=W*\tan \alpha \end{aligned}$$

If $\alpha$ greater than $\frac{\pi }{4}$:

17 $$\begin{aligned} w1=\frac{H}{\tan \alpha },h1=H \end{aligned}$$

Based on the above calculations of *w*1 and *h*1, the final calculated *C* is as follows:

18 $$\begin{aligned} C=\sqrt{\left( h1^2+w1^2\right) } \end{aligned}$$

Although the improved definition of *C* accelerates the convergence of the bounding box, when the predicted bounding box is very close to the ground truth but does not overlap, the gradient contributed by the penalty term will decrease significantly, making convergence difficult. To address this issue, we introduce a scale factor into the loss function. The definition formula for scale is as follows, where “downsample” represents the downsampling ratio of the current detection feature map. For example, when the input image size is 640 $\times$ 640 and the detection feature map size is 80 $\times$ 80, the “downsample” is 8 and the scale is 3.

19 $$\begin{aligned} scale=\log _{2}{\left( downsample \right) } \end{aligned}$$

This scale factor ensures that the penalty term continues to provide a substantial gradient even when the two bounding boxes are very close to each other, effectively overcoming convergence bottleneck. Note that the scale factor serves as a constant to amplify the gradient, and its calculation process is excluded from the gradient computation related to the penalty term. The final loss function is defined as follows.

20 $$\begin{aligned} S\text {-}MPDIoU=IoU-scale*\left( \frac{d_1^2}{C^2}-\frac{d_2^2}{C^2}\right) \end{aligned}$$

### MDA

Conventional attention mechanism typically focus on feature information in two dimensions: channel and spatial. This lead to diverse design concepts, ranging from average pooling to directional pooling that captures features in both width and height dimensions, introducing positional information. Furthermore, multi-scale feature interaction enables the flow of information between feature maps of different sizes, enhancing overall feature representation capability. However, these techniques often use channel grouping and channel shuffle to save parameters. This leads to the loss of input feature information and increases the complexity of the model, rendering such techniques unsuitable for some scenarios.

**Fig. 4:**
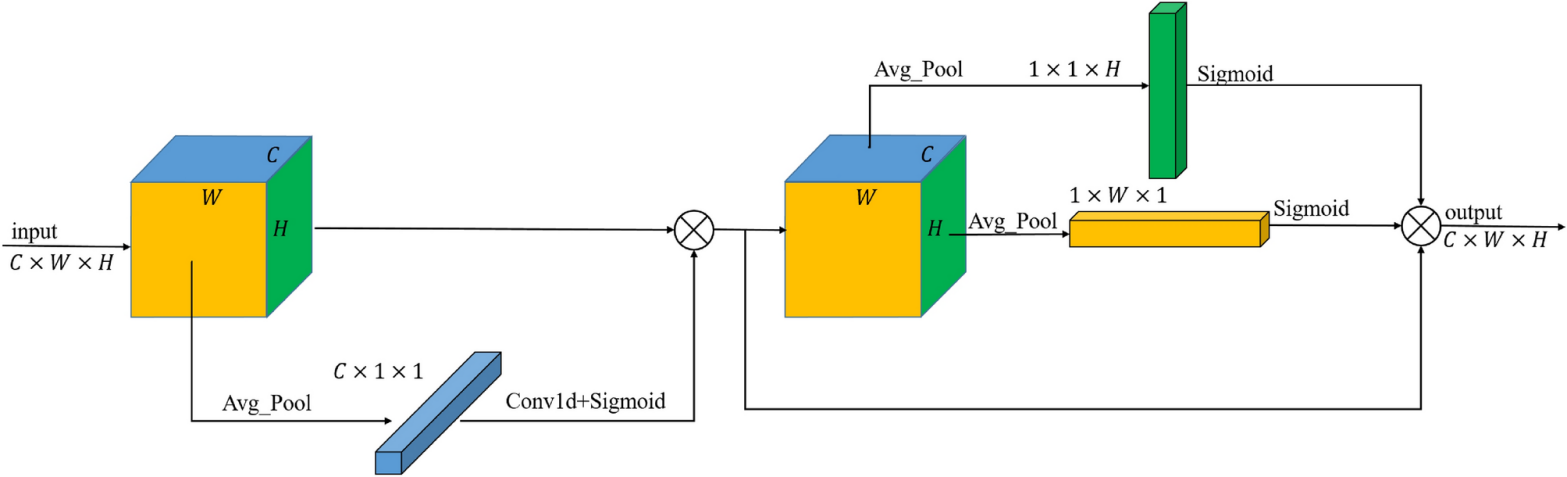
MDA module. Orange, green, and blue are used to represent the feature information of width, height, and channel, respectively.

We propose a simple and effective attention mechanism, named Multi-Dimensional Attention (MDA). MDA effectively enhances the detection performance of the model while reducing the parameters. The schematic diagram of MDA is presented in Fig. 4. Channel attention mechanisms typically perform feature extraction in the spatial dimension, whereas spatial attention mechanisms extract features in the channel dimension. However, the width and height feature correlation information among channels also holds significant value.

Capturing cross-channel width and height information further enriches and enhances the extracted features. This approach establishes a close relationship between each pixel in the feature map and the global context, rather than relying on a single dimension. The lymphocyte dataset used in this paper has numerous small-sized objects and a complex background with various distractions. Conventional attention mechanisms can easily overlook important features. Therefore, adopting multi-dimensional feature extraction can more effectively retain valuable information and improve detection accuracy.

Let the input size be C $\times$ W $\times$ H . Input initially undergoes average pooling along the spatial dimension to extract feature information, resulting in a feature vector of size C $\times$ 1 $\times$ 1, which is roughly the same as conventional channel attention. Then we apply adaptive 1D convolution to the feature vector, facilitating cross-channel information exchange and integrating channel correlations. After nonlinear processing through the Sigmoid activation function, these sets of statistics are multiplied by their corresponding elements of the input to obtain the refined feature map.

Then, average pooling is applied to the width and height dimensions of the newly obtained feature map for feature extraction. The feature vector obtained from the width dimension has a size of 1 $\times$ W $\times$ 1, indicating that it captures cross-channel height information and reflects it in the width dimension. Similarly, the feature vector from the height dimension has a size of 1 $\times$ 1 $\times$ H, capturing cross-channel width information and reflecting it in the height dimension. These two dimensional feature vectors are then nonlinearly processed through the Sigmoid activation function. Finally, these sets of statistics are multiplied by their corresponding elements of input to obtain the output. MDA, with the above design strategy, can fuse multi-dimensional features, resulting in benefits for the detection of small and medium-sized objects, while having significantly fewer parameters compared to other attention mechanisms.

## Experiment and analysis

### IoU loss function simulation experiment

#### Simulation experiment 1

To evaluate the performance of S-MPDIoU compared to other IoU loss functions, we conduct simulation experiment 1. Different IoU loss functions exhibit high sensitivity in adjusting the learning rate, so even small changes in the learning rate may lead to significant differences in the final convergence effect. To ensure a more equitable evaluation, the learning rate is fixed at 0.02. The choice of this small learning rate value is carefully considered, as it enables all IoU loss functions to converge while minimizing the impact of learning rate changes on the final result.

Assuming the four parameters of the ground truth bounding box are $\left( x^{gt},y^{gt},w^{gt},h^{gt} \right)$, the four parameters of the predicted bounding box are $\left( x,y,w,h\right)$, the loss defined in this simulation experiment is as follows:

21 $$\begin{aligned} Loss=\left| x^{gt}-x \right| +\left| y^{gt}-y \right| +\left| w^{gt}-w \right| +\left| h^{gt}-h \right| \end{aligned}$$

**Fig. 5:**
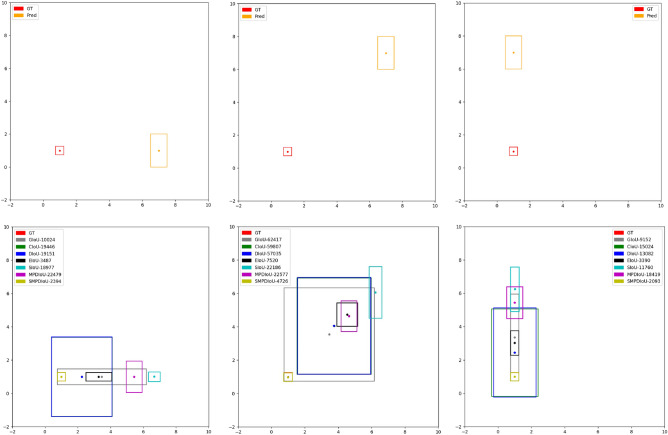
Diagram of the simulation experiment 1. The diagram depicts three cases: horizontal, diagonal, and vertical, arranged from left to right. The ground truth bounding box center coordinates are fixed at [1, 1], with both width and height set to 0.5. The predicted bounding box has a fixed width of 1 and a height of 2, with center coordinates of [7, 1], [7, 7], and [1, 7], respectively.

The training epochs are set to 100,000. A predicted bounding box is considered converged when the loss is less than 0.01. The specific experimental results are shown in Fig. 5. The experiment examines the convergence effects from three different cases: horizontally aligned boxes, diagonally aligned boxes, and vertically aligned boxes. The legend indicates the different IoU loss functions and the epochs needed to achieve convergence. Notably, in Fig. 5, S-MPDIoU achieves convergence in all three cases.

As seen in Fig. 5, the initial predicted bounding boxes are larger in size compared to the ground truth bounding box. The convergence processes of DIoU and CIoU exhibit distinct shape changes. Initially, they tend to enlarge the dimensions of the predicted bounding box and then shift the box towards the ground truth. Once the predicted bounding box encompasses the ground truth, DIoU and CIoU will shrink its dimensions to achieve convergence.

On the other hand, EIoU and SIoU initially shrink the width and height of the predicted bounding box. As one or both of the dimensions (width or height) converge, the predicted bounding box gradually moves closer to the ground truth. Notably, EIoU exhibits significantly faster convergence than SIoU. This difference can be attributed to the complexity of SIoU’s penalty term, which potentially introduces additional interference, thereby slowing down the convergence process.

The convergence of GIoU is more challenging and unstable. In the diagonal case, GIoU behaves similarly to DIoU. In the vertical and horizontal cases, GIoU behaves similarly to EIoU. However, these convergence processes are all very slow.

MPDIoU does not exhibit significant deformation in all three cases. Its convergence trend is initially to reduce the distance, and then to adjust dimensions. However, when the predicted bounding boxes are very close to the ground truth yet do not overlap, IoU does not contribute to the gradient calculation, and the gradient contribution from MPDIoU’s penalty term diminishes sharply, leading to a notably slow convergence process.

S-MPDIoU exhibits a convergence trend analogous to MPDIoU. In the diagonal case, where the centers of the predicted and ground truth bounding box form a 45-degree angle, the baseline length remains identical to that of MPDIoU. However, the introduction of scale factor enables S-MPDIoU to sustain a considerable gradient, even when the predicted bounding box is close but does not overlap with the ground truth bounding box, ultimately achieving a convergence speed almost 6 times faster. In the vertical and horizontal cases, the baseline in S-MPDIoU penalty term takes into account the angle between the centers of the two bounding boxes, further improving convergence speed by almost 10 times.

Based on the simulation experiment, it is clearly demonstrated that S-MPDIoU preserves the strengths of MPDIoU, effectively preventing abrupt shape variations and wandering during the training process. In each epoch, it consistently converges along a fixed direction between the two bounding boxes. By introducing the scale factor and redefining the penalty term, S-MPDIoU overcomes the problem of gradient diminution encountered by MPDIoU, leading to a marked improvement in convergence speed.

#### Simulation experiment 2

**Fig. 6:**
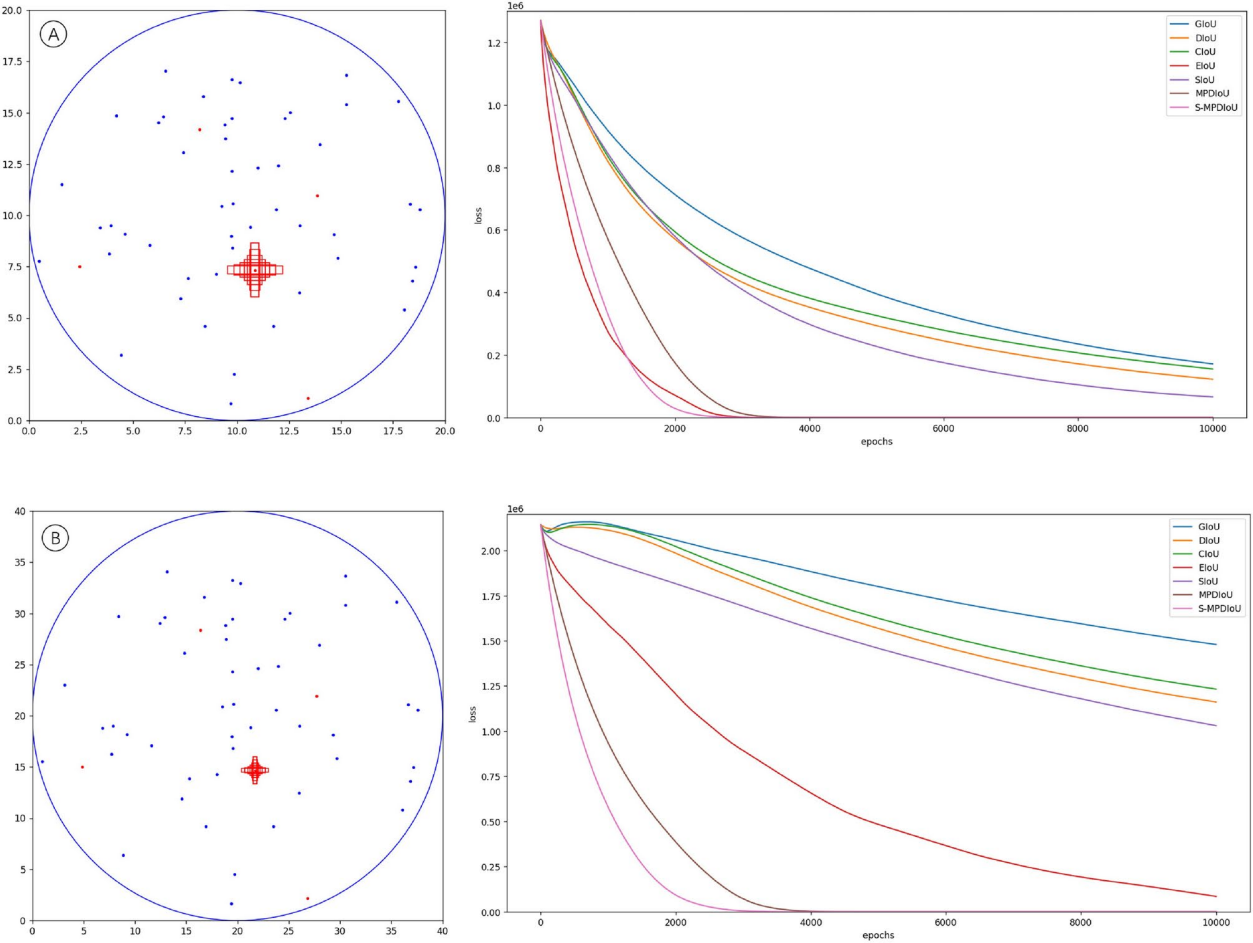
Schematic diagram of simulation experiment 2. (**A**) simulates the 20 $\times$ 20 feature map and (**B**) simulates the 40 $\times$ 40 feature map. The right figures show the convergence performance of different IoU loss functions.

To further evaluate the convergence performance of different IoU loss functions, we conduct simulation experiment 2. In Fig. 6, A and B simulate feature maps of sizes 20 $\times$ 20 and 40 $\times$ 40, right figures show the convergence effect of different IoU loss functions. Inside circles with radii of 10 and 20, respectively, 50 center coordinates of predicted bounding boxes are randomly generated and represented as blue dots. Additionally, 5 center coordinates of ground truth bounding boxes are randomly generated and represented as red dots. Each dot corresponds to 7 bounding boxes with varying aspect ratios, shown as red boxes in the left figures. These boxes maintain an area of 1, with aspect ratios of 7:1, 4:1, 2:1, 1:1, 1:2, 1:4, and 1:7, respectively. Furthermore, each predicted bounding box has 7 additional scales: 0.5, 0.67, 0.75, 1, 1.33, 1.5, and 2. Therefore, the total number of regression cases is 89,500 = 50 $\times$ 7 $\times$ 7 $\times$ 5 $\times$ 7.

To reduce the complexity of the experiment and accelerate the convergence, the learning rate is fixed at 0.02. A predicted bounding box is considered converged when the loss is less than 0.5. Training epochs are set to 10,000.

Figure 6A simulates the 20 $\times$ 20 feature map. During the early stage of training, since the predicted bounding boxes are situated relatively close to the ground truth, EIoU effectively adjusts the width and height of the predicted bounding boxes, leading to a swift reduction in the calculated loss. However, in the later stage of training, due to insufficient gradient contribution from its penalty term for the distance, its convergence speed begins to slow down. On the other hand, S-MPDIoU maintains a stable convergence trend and converges earlier than EIoU in the end.

Figure 6B simulates the 40 $\times$ 40 feature map. In this case, the distance between the predicted and ground truth bounding box is further increased, resulting in a significant increase in the difficulty of convergence for loss functions such as CIoU, DIoU, and GIoU, which rely more on IoU. In the early stage of training, these IoU loss functions constantly enlarge the width and height of the predicted bounding box, but the movement cannot compensate for the loss caused by the shape change, leading to an increase in the total loss. These IoU loss functions begin to converge as the deformation of the predicted bounding box gradually tapers off and ultimately comes to a halt. However, due to the large distance between the predicted and ground truth bounding box and the insufficient gradient contribution from the distance penalty term, their convergences are very slow.

MPDIoU’s advantages are especially apparent when the distance between the predicted and the ground truth bounding box is relatively large. Its penalty term significantly contributes to the gradient, allowing the predicted bounding box to move quickly towards the ground truth during the early stage of training. However, once the predicted bounding box approaches the ground truth, it still faces the problem of a sharp decrease in gradient, resulting in a slowdown in convergence speed. Nevertheless, its overall convergence speed is superior to other IoU loss functions mentioned above. Although EIoU converges relatively quickly, its convergence also slows down due to the distance. In contrast, S-MPDIoU consistently maintains a faster convergence speed, demonstrating a significant advantage.

### Cell detect experiment

#### Experimental environment and parameter settings

The original YOLOv8 algorithm provides five different scale models: N, S, M, L, and X. Although the structure of these five scale models remains same, each scale model has different depths and widths, resulting in different sizes and complexities. In this paper, we test and analyze the YOLOv8n’s ability to detect lymphocytes in experiments.

The platforms used for model training in this experiment are Intel Core i9-13900 CPU and NVIDIA GTX4060 8G GPU. The software uses the Windows system, Python 3.11, PyTorch 2.0.1, and Cuda11.8 deep learning framework. The implementation uses libraries like torch, torchvision, pyyaml, opencv, matplotlib, and Numpy.

The training epochs are set to 100 with a batch size of 6. The input image size is 640 $\times$ 640. The initial learning rate is set to 0.001, and ADAM is used as the optimization algorithm. The weight decay is 0.005, and the momentum is set to 0.937. All default data augmentation methods have been deactivated.

#### Experimental dataset

**Fig. 7:**
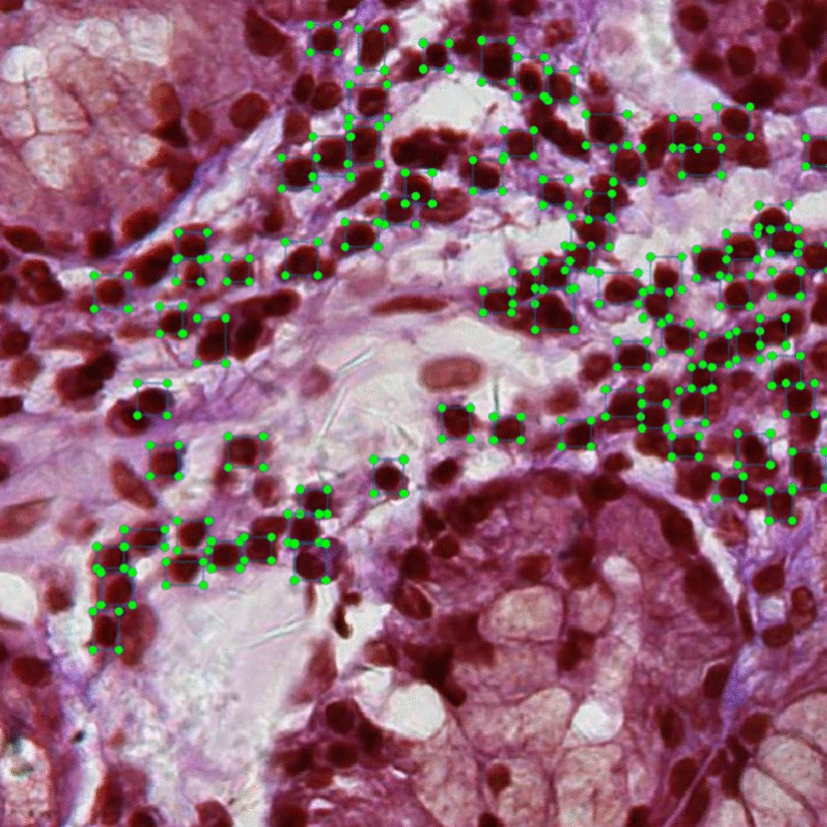
Illustration for manually annotating dataset. Green boxes indicate the manually annotated lymphocytes.

The experimental dataset used in this paper is sourced from WSI of labial gland biopsy specimens from YanTaiShan Hospital. The use of this dataset has been approved by the Hospital Ethics Review Committee. Due to the large size of WSI, direct detection is not feasible. Therefore, we segment the original WSI into patches with a size of 640 $\times$ 640 at the highest resolution, and create a dataset containing 600 images under the manual screening and annotation of professional physicians. It should be noted that the average number of lymphocytes contained in a single image exceeds 30, which makes the manual labeling process extremely cumbersome and time-consuming. The sole detection object is lymphocyte, and there are a significant number of lymphocytes with distinctive and consistent features present in a single image. So we manually annotate 600 images and choose a lighter model for training to achieve a balance between model training and annotation complexity. The dataset is divided into training, validation, and testing sets in 8:1:1. An example of annotated images is shown in Fig. 7, where the green boxes represent manually annotated lymphocytes.

#### Evaluation indicators

We utilize a set of standard metrics to evaluate the performance of the improved YOLOv8n in lymphocyte detection tasks. The primary metrics considered in this paper are Recall (R), Precision (P), and mean Average Precision (mAP). Since the sole object to be detected in our dataset is lymphocyte, these metrics can be represented as follows:

22 $$mAP = \int_{0}^{1} P \left( R \right)\left\lceil R \right.$$

23 $$\begin{aligned} & P=\frac{TP}{\left( TP+FP \right) } \end{aligned}$$

24 $$\begin{aligned} & R=\frac{TP}{\left( TP+FN\right) } \end{aligned}$$

Among these metrics, TP (true positive) refers to instances that are correctly predicted as positive, TN (true negative) refers to instances that are correctly predicted as negative, FP (false positive) refers instances that are incorrectly predicted as positive, and FN (false negative) refers instances that are incorrectly predicted as negative.

#### Experimental results

We first evaluate the performance of different IoU loss functions, and the experimental results are presented in Table 1. As can be seen from Table 1, S-MPDIoU has the highest mAP among all IoU loss functions, and its precision and recall are also stable. Compared with the CIoU of YOLOv8, its detection precision slightly reduces by 0.7%, but the recall increases by 7.8%, mAP.5 increases by 4.3%, and mAP.95 increases by 2.1%. The experimental results fully demonstrate the effectiveness of S-MPDIoU.

**Table 1:** Detection performance of different IoU loss functions.

Algorithm	P%	R%	mAP.5%	mAP.95%
V8n+CIoU	80.4	77	85.3	35.9
+DIOU	78.6	84.7	87.8	36.8
+GIOU	81.6	84	88.1	38
+EIOU	77.1	82.3	85.7	34.6
+SIOU	77.4	85.5	86.8	36.7
+MPDIOU	77.7	85.6	88.4	37.7
+S-MPDIOU	79.7	84.8	89	38

Then we conduct an experiment to compare and analyze the detection performance of different attention mechanisms. The results are shown in Table 2. Compared with SE, CBAM, GAM, CA, ECA, EMA, and SA, the MDA module has slightly lower recall, but higher precision and mAP, with relatively fewer parameters. Its detection precision increases by 0.5%, recall increases by 6.3%, mAP.5 increases by 3.6%, and mAP.95 increases by 1.9%. This experiment proves that integrating MDA modules into the backbone of the detection model helps enhance and fuse features, achieving an effective balance between detection accuracy and parameter efficiency.

**Table 2:** Detection performance of different attention mechanisms.

Algorithm	P%	R%	mAP.5%	mAP.95%	Parameters
V8n	80.4	77	85.3	35.9	3.006 $$\times$$$$10^6$$
+SE	79.8	84.6	88.3	37.8	3.017 $$\times$$$$10^6$$
+CBAM	80.3	85.2	89.1	37.2	3.094 $$\times$$$$10^6$$
+GAM	78	86.1	87.9	37.9	3.585 $$\times$$$$10^6$$
+CA	78.2	84.8	86.5	36.2	3.018 $$\times$$$$10^6$$
+ECA	78.8	85.8	88.1	38.2	3.006 $$\times$$$$10^6$$
+EMA	76.7	85.8	87	36.8	3.006 $$\times$$$$10^6$$
+SA	79.4	80.6	86.2	37.1	3.006 $$\times$$$$10^6$$
+MDA	80.9	83.3	88.9	37.8	3.006 $$\times$$$$10^6$$

**Fig. 8:**
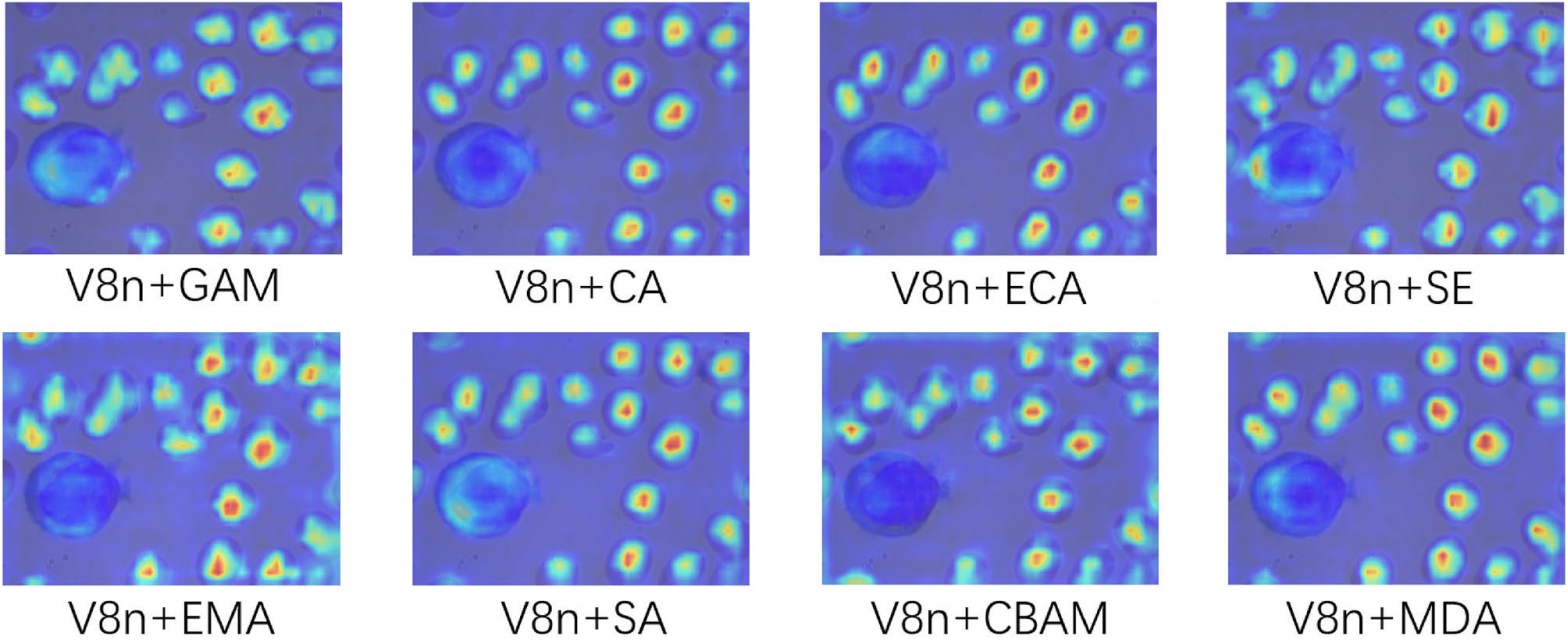
Heatmaps of different attention mechanisms.

The heatmaps presented in Fig. 8 demonstrate the efficacy of different attention mechanisms. As shown in the figure, the attention area of MDA is smoothly distributed and comprehensive, evidently focusing on the prominent features of cells while overcoming background interference that would distract attention. Even the cell features in the edge area can be effectively captured. Further demonstrate the advantages of the MDA module.

Afterwards, we conduct an ablation experiment to evaluate the effectiveness of these improvements. As shown in Table 3, after integrating the above improvements into the yolov8n, the final precision of the model reaches 80%, a relative decrease of 0.4%. However, the recall reaches 86.1%, a significant increase of 9.1%. In addition, mAP. 5 reaches 88.5%, a relative increase of 3.2%, and mAP. 95 reaches 37.9%, a relative increase of 2%. These results confirm the effectiveness of the improvements proposed in this paper.

**Table 3:** Results of ablation experiment.

S-MPDIoU	MDA	P%	R%	mAP.5%	mAP.95%
$\times$	$\times$	80.4	77	85.3	35.9
$\checkmark$	$\times$	79.7	84.8	89	38
$\times$	$\checkmark$	80.9	83.3	88.9	37.8
$\checkmark$	$\checkmark$	80	86.1	88.5	37.9

We conducted an exhaustive comparative experiment to rigorously evaluate the performance of our improved YOLOv8 model against several state-of-the-art models with similar parameter settings and capabilities, including YOLOv9t, YOLOv10n, RT-DETR^27^, GOLD-YOLO^28^, and PP-YOLOE^29^. The results of this experimentation, presented in Table 4, offer evidence of the balance our model achieves in terms of detection accuracy and complexity.

Specifically, our improved YOLOv8 model demonstrated a notable increase in mean average precision (mAP) compared to YOLOv9t and YOLOv10n. This enhancement in accuracy is particularly significant given the competitive nature of these models and underscores the effectiveness of our proposed improvements. Furthermore, when compared to the transformer-based RT-DETR, our model achieved comparable accuracy. In addition, while the experimental results indicate that our model’s accuracy is marginally lower than the powerful GOLD-YOLO, it significantly reduces the parameters and complexity, underscoring its proficiency in striking an effective balance between performance and efficiency. Similarly, when compared to the highly optimized PP-YOLOE, our model demonstrated competitive results, demonstrating its robustness and adaptability across different optimization strategies.

**Table 4:** Algorithm comparison results.

Algorithm	mAP.5%	mAP.95%	Parameters	GFLOPs
YOLOv8n	85.3	35.9	3.006 $$\times$$$$10^6$$	8.1
YOLOv8s	84.3	37.4	11.126 $$\times$$$$10^6$$	28.4
YOLOv9t	84.6	34	2.617 $$\times$$$$10^6$$	10.7
YOLOv10n	83	35.5	2.265 $$\times$$$$10^6$$	6.5
RT-DETR	84.4	36.4	20 $$\times$$$$10^6$$	60
GOLD-YOLO	89.1	38.8	5.976 $$\times$$$$10^6$$	10.2
PP-YOLOE+-S	83.2	34.1	7.36 $$\times$$$$10^6$$	17.93
OURS	88.5	37.9	3.006 $$\times$$$$10^6$$	8.1

**Fig. 9:**
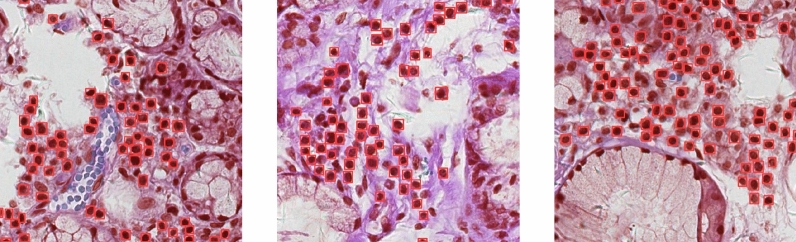
Detection effect of our improved model, where the red boxes represent the lymphocytes detected by the model.

The detection effect of our improved model is shown in Fig. 9, where the red boxes represent the lymphocytes detected by the model. As shown in the figure, the improved model fully learns the characteristics of lymphocytes, follows the criteria for lymphocyte discrimination, and overcomes the interference caused by complex backgrounds. For suspicious similar cells such as epithelial cells and plasma cells, it basically achieves correct discrimination and completes the detection and localization task.

### Auxiliary diagnostic system for Sjogren’s syndrome

**Fig. 10:**
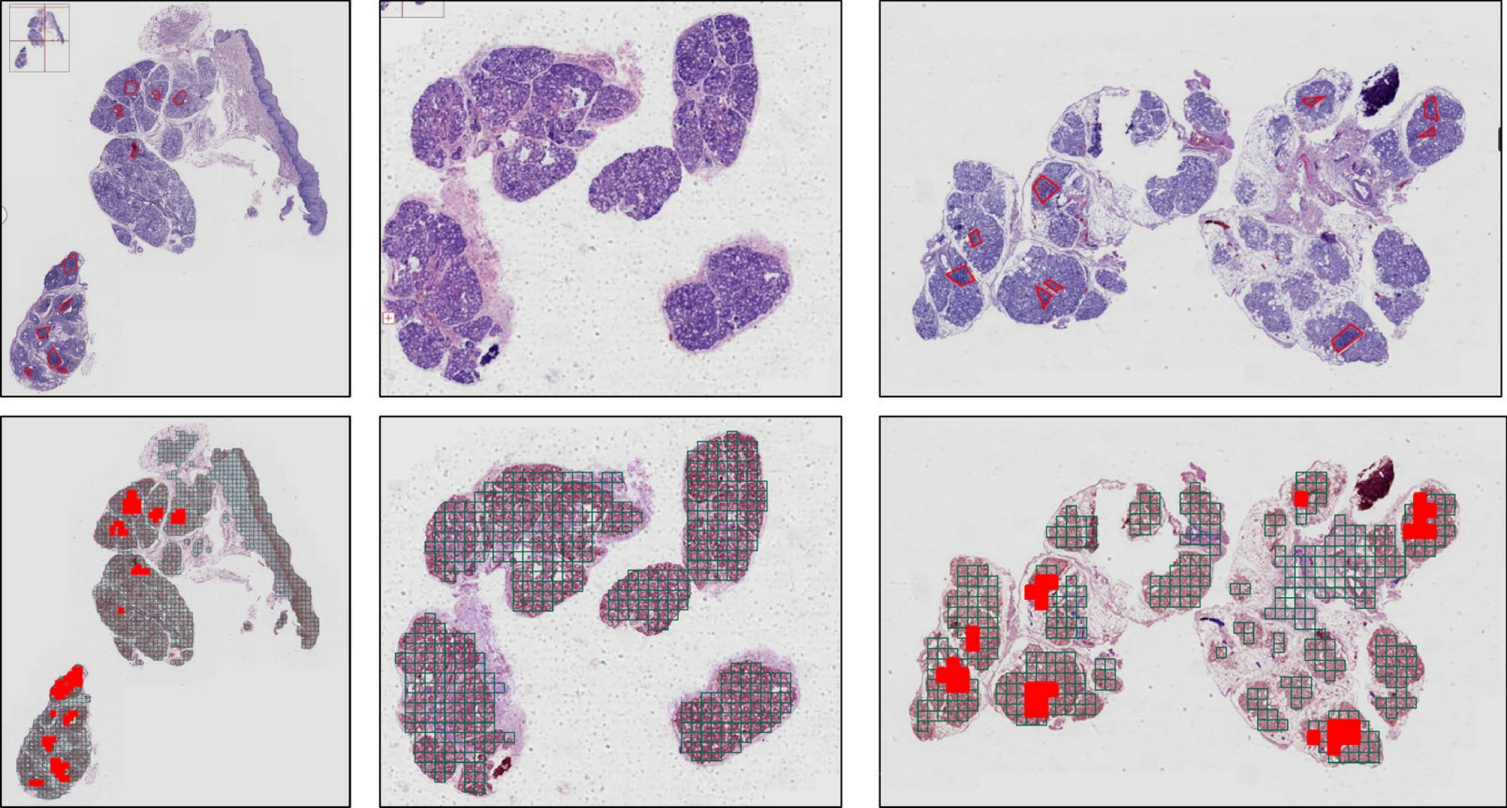
Effect of auxiliary diagnostic system. Top three images represent the lesions annotated by the physician, and the bottom three images represent the lesions identified by auxiliary diagnostic system.

As presented in Fig. 10, the top three images represent the lesions annotated by the physicians, while the bottom three images represent the lesions identified by auxiliary diagnostic system. It is evident that the system effectively identifies and labels suspicious lesions, which roughly correspond to those annotated by the physicians, indicating that the system boasts high accuracy in lesion discernment and can assist in pathological diagnosis to a certain extent.

### Limitations

From the experimental results, it is evident that our designed diagnostic system has attained a high level of detection accuracy, thereby partially fulfilling the objective of assisting physicians in diagnosing Sjogren’s syndrome. Nevertheless, the system still possesses certain limitations. Firstly, due to the small number of datasets, the model may not have fully learned the underlying patterns and relationships within the data. This limitation can lead to underfitting, where the model fails to capture the complexity of the data and is unable to generalize well to new, unseen examples. As a result, the model’s performance may be suboptimal, and it may struggle to make accurate predictions or classifications. Secondly, as the system directly performs pathological diagnosis on pathological images, and the preparation of actual pathological images involves multiple steps, including specimen collection, processing, staining, and digitalization, the practical application of the system in clinical practice still requires careful validation and integration into existing workflows to ensure accuracy, reliability, and efficiency in patient care.

## Conclusion

In recent years, the application of deep learning technology in the medical field has progressed rapidly, significantly contributing to the diagnostic process of physicians through its remarkable efficiency and accuracy. The manual diagnosis of Sjogren’s syndrome, however, remains time-consuming and labor-intensive, requiring physicians to meticulously discern lymphatic infiltration in pathological sections, ultimately leading to low diagnostic efficiency. To tackle this challenge, we devised an approach utilizing deep learning techniques to assist in the diagnostic process. Specifically, we developed an auxiliary diagnostic system leveraging the state-of-the-art object detection network, YOLOV8. This system initially segments the original pathological image into multiple patches, which are then individually fed into the detection network. Subsequently, the individual detection results are aggregated to yield the final diagnostic outcome. To refine the network’s detection accuracy, we introduced the multi-dimensional attention mechanism and S-MPDIoU loss function. The multi-dimensional attention mechanism effectively captures cellular features, enhancing detection accuracy by separately extracting features from the three dimensions of the feature map. Additionally, the S-MPDIoU loss function mitigates the issue of gradient vanishing between ground truth and predicted bounding boxes during training, thereby enhancing both training efficiency and detection accuracy.The precision of the improved model decreases by 0.4%, the recall increases by 9.1%, mAP.5 increases by 3.2%, and mAP.95 increases by 2%. These results prove the effectiveness of the improvements.

Our research presents novel diagnostic tools and algorithms that offer fresh insights into Sjogren’s syndrome diagnosis. Through the development and refinement of diagnostic tools, our study has achieved a notable accuracy of Sjogren’s syndrome diagnosis. This enhanced accuracy empowers physicians to make more informed decisions, facilitating earlier interventions and ultimately leading to better patient outcomes.Our research is attributed to the valuable collaborations with experts from diverse fields, including medicine and computer science. This interdisciplinary approach has enabled us to integrate diverse perspectives and expertise, leading to the development of more comprehensive and effective diagnostic solutions for Sjogren’s syndrome. Our study not only provides important insights into Sjogren’s syndrome diagnosis but also lays a solid foundation for future research.

However, two main limitations were identified: the limited size of the datasets, which could result in underfitting and suboptimal model performance, and the complex practical application process of the system involving pathological image preparation, requiring careful validation and integration into clinical workflows.Future research should focus on expanding pathological image datasets using advanced data augmentation, conducting comprehensive clinical validation to ensure seamless integration into workflows, and exploring multi-modal fusion approaches that integrate additional data sources beyond images to enhance diagnostic accuracy and comprehensiveness.

## Data Availability

The data used to support the findings of this study is available from the corresponding author upon request.
